# Laparoscopic Colorectal Surgery in Resident Training: Is It Safe and Feasible?

**DOI:** 10.3390/healthcare14183076

**Published:** 2026-09-18

**Authors:** Göksever Akpınar, Olcay Atbaş, Safa Vatansever, Batuhan Eyduran, Yüksel Çalık, Ozan Barış Namdaroğlu, Kamil Pehlivanoğlu

**Affiliations:** 1 Department of General Surgery, University of Health Sciences Tepecik Training and Research Hospital, Izmir 35020, Türkiye; gokseverakpinar@gmail.com (G.A.); ozannamdaroglu@yahoo.com (O.B.N.); kamil.pehlivanoglu@saglik.gov.tr (K.P.); 2 Department of General Surgery, Faculty of Medicine, Ege University, Izmir 35040, Türkiye; safavatansever@gmail.com; 3 Department of General Surgery, Beypazarı State Hospital, Ankara 06730, Türkiye; batuhaneyduran@yahoo.com.tr; 4 Department of General Surgery, Buyukcekmece Mimar Sinan Hospital, Istanbul 34535, Türkiye; yukselcalik199595@gmail.com

**Keywords:** laparoscopic colorectal surgery, resident training, surgical education, patient safety, oncologic outcomes

## Abstract

**Background/Objectives**: The evolution of minimally invasive techniques has established advanced laparoscopic skills as an increasingly essential component of surgical curricula. However, the feasibility of senior residents performing technically demanding procedures such as Laparoscopic Colorectal Surgery (LCS) remains debated. This study evaluated the perioperative safety, short-term oncologic outcomes, and operative-time implications of supervised Laparoscopic Colorectal Surgery (LCS) performed by senior residents (post-graduate years 3 to 5 of a 5-year program). **Methods**: A retrospective observational cohort study of 136 patients undergoing curative-intent LCS for primary colorectal malignancy was conducted. Patients were stratified into a Specialist group (*n* = 102) and a Resident group (*n* = 34). Residents were senior trainees operating under the direct intraoperative supervision of experienced specialists. Perioperative metrics, patient safety endpoints, and short-term oncologic outcomes were compared. **Results**: The groups were demographically and pathologically well-matched, demonstrating similar distributions of advanced-stage (T3–T4) tumors and high Body Mass Index. Median operative time was significantly prolonged in the Resident group (340 vs. 290 min; *p* = 0.023). However, no significant differences were observed regarding the median length of hospital stay (9 vs. 8 days; *p* = 0.057), the incidence of anastomotic leakage (3.4% vs. 3.8%; *p* = 1.000), or positive surgical margin rates (5.9% vs. 10.8%; *p* = 0.517). No statistically significant difference in median lymph node yield was detected between the Resident and Specialist cohorts (17 vs. 16 nodes; *p* = 0.251). **Conclusions**: Under direct specialist supervision, resident-performed LCS was associated with no material difference in the measured short-term outcomes within this single-center supervised training model and the eligibility criteria used. The prolongation of operative time associated with resident participation appears to be a safe and acceptable aspect of surgical training.

## 1. Introduction

In recent years, a distinct shift towards minimally invasive techniques has been observed in general surgery practice worldwide. This transformation has reshaped approaches to operating room safety, operational efficiency, and surgical education. High-level evidence presented by Lacy et al. [1] and the COST trial [2] demonstrated that the laparoscopic approach offers less postoperative pain and faster recovery compared to open surgery, while maintaining similar oncologic outcomes [3]. These and similar studies have facilitated the widespread adoption of minimally invasive surgical techniques, establishing Laparoscopic Colorectal Surgery (LCS) as the gold standard for the treatment of both benign and malignant diseases [1,2,3]. This technological and procedural evolution necessitates the integration of minimally invasive surgery into training programs.

Today, basic procedures such as laparoscopic cholecystectomy and appendectomy have become integral parts of the general surgery residency curriculum. However, traditional training models are increasingly being replaced by structured, proficiency-based curricula due to patient safety concerns and the growing complexity of modern surgery. The integration of advanced laparoscopic procedures—such as Laparoscopic Colorectal Surgery, which requires multi-quadrant work and possesses a steep learning curve—into resident training remains a major topic of contemporary surgical education [4,5]. Recent literature highlights that by employing a stepwise approach to training, even technically formidable operations such as laparoscopic surgery for complex Crohn’s disease can be safely performed by supervised trainees [6]. The question of whether advanced laparoscopic procedures can be performed by residents with safety and efficacy comparable to experienced surgeons lies at the center of the delicate balance between “educational quality” and “patient safety”. While recent studies in the literature emphasize that structured resident participation does not increase morbidity [7], historical concerns regarding the relationship between experience and long-term oncologic outcomes are increasingly being addressed. For instance, recent propensity-score matched analyses have suggested that supervised training may not adversely affect short-term surgical safety or long-term oncological endpoints, including 5-year overall and disease-free survival [8]. In this context, while current literature provides valuable insights, evidence remains mixed depending on trainee level and case selection. Therefore, it is essential to further clarify whether therapeutic efficacy is compromised while residents gain competence in the training process of Laparoscopic Colorectal Surgery, one of the most complex procedures evaluated within this scope.

This study aims to contribute to this specific gap in the literature by evaluating the feasibility of Laparoscopic Colorectal Surgery performed by senior general surgery residents (post-graduate year 3 and above) under the supervision of experienced general surgery specialists; and to examine the effects of this process on patient safety, perioperative outcomes, and short-term oncologic adequacy.

## 2. Materials and Methods

### 2.1. Study Design and Patient Collection

This is a single-center, retrospective, observational cohort study analyzing the prospectively maintained institutional database of patients who underwent curative-intent laparoscopic colorectal resections for primary colorectal malignancies at the Department of General Surgery, Izmir Tepecik Training and Research Hospital, between January 2020 and December 2024. Data were entered into the electronic database by trained surgical fellows and were routinely audited for completeness and accuracy. The audits were performed independently on a monthly basis by a senior researcher not involved in the initial data entry process.

### 2.2. Data Extraction and Clinical Parameters

Demographic, clinicopathological, perioperative, and postoperative data were systematically extracted from electronic health records for each individual case. The recorded clinical parameters and analyzed variables included patient baseline characteristics (age, gender, body mass index [BMI], and the presence of comorbidities), pathological tumor staging (pathological T and N classifications), total operative time (minutes), total number of harvested lymph nodes, surgical margin status (positivity vs. negativity), postoperative length of hospital stay (days), and short-term mortality rates. Regarding postoperative morbidity, complications were specifically monitored and documented for the development of anastomotic leakage. The specific types of surgical procedures performed were categorized as follows: right hemicolectomy, left hemicolectomy, anterior resection (AR), low anterior resection (LAR), abdominoperineal resection (Miles’ procedure), and subtotal/total colectomy.

### 2.3. Operative Cohorts and Training Paradigm

All surgical procedures were performed utilizing a standardized totally laparoscopic approach. To evaluate the impact of the institutional training paradigm on surgical outcomes, the cohort was stratified into two groups based on the status of the primary operating surgeon.

1. Specialist Group: Procedures performed independently by a general surgery specialist.
2. Resident Group: Patients operated on by resident doctors under the direct supervision of an experienced surgeon.

Residents were defined as “senior general surgery trainees” (post-graduate year 3 and above, specifically representing the 3rd, 4th, and 5th years of a 5-year training program) in accordance with the Turkish Surgical Society training curriculum. These residents had previously achieved proficiency in basic laparoscopic procedures (e.g., cholecystectomy, appendectomy) and had served as first assistants in a standardized minimum number of complex colorectal cases. A procedure was defined as ‘resident-performed’ if the resident completed the critical key steps of the operation (e.g., major vascular ligation, splenic flexure mobilization, and anastomosis) as the primary operator. Furthermore, case allocation to the residents was determined by their scheduled clinical rotations rather than the perceived simplicity of the case. All resident cases were directly supervised by one of three experienced colorectal surgery specialists, who were required to have at least 7 years of experience as a specialist and to have performed a total of at least 150 Laparoscopic Colorectal Surgery cases.

### 2.4. Exclusion Criteria

To mitigate case-mix heterogeneity and control for patient-related surgical complexity, the following exclusion criteria were rigorously applied:

- Cases requiring conversion from laparoscopic to open surgery;
- Patients operated on for non-malignant (benign) indications;
- Patients diagnosed with metastatic colorectal cancer;
- Patients with a history of surgery for a prior malignancy;
- Patients with a history of prior intra-abdominal surgery.

### 2.5. Statistical Analysis

Statistical processing and analyses were executed using SPSS software (Version 26.0; IBM Corp., Armonk, NY, USA). The normality of distribution for continuous variables was assessed using the Shapiro–Wilk test and visual inspection of Q-Q plots. Normally distributed continuous variables are reported as mean ± standard deviation (SD) and were compared using the independent samples Student’s *t*-test. Non-normally distributed variables are expressed as median with range (minimum-maximum) and were analyzed via the Mann–Whitney U test. Categorical variables are presented as frequencies and percentages (%). Inter-cohort comparisons for categorical data were performed using the Pearson Chi-square test, Fisher’s exact test, or the Likelihood Ratio test, as appropriate. A two-sided *p*-value of <0.05 was defined as the threshold for statistical significance.

Due to the low number of clinical events for specific complications such as anastomotic leakage, multivariable regression analysis was not performed to avoid statistical overfitting. Therefore, the outcomes were evaluated utilizing descriptive and univariate inferential statistics.

## 3. Results

### 3.1. Study Population and Baseline Homogeneity

A total of 136 patients who met the predefined inclusion criteria were included in the final analysis. The cohort was stratified into the Specialist group (*n* = 102, 75%) and the Resident group (*n* = 34, 25%). No statistically significant differences were observed in the baseline demographic and clinical characteristics between the two cohorts. There were no statistically significant differences in the mean age (61.8 ± 10.7 vs. 62.2 ± 11.8 years; mean difference 0.40 years, 95% CI −4.18 to 4.98; *p* = 0.868) or the median Body Mass Index (26.33 vs. 26.37 kg/m^2^; *p* = 0.629) (Table 1). Although the proportion of female patients was numerically higher in the Resident group, the gender distribution remained statistically comparable (*p* = 0.115) (Table 1).

### 3.2. Procedural Characteristics and Tumor Pathology

The distribution of the specific surgical procedures was similar between the cohorts (*p* = 0.582), with Low Anterior Resection (LAR) being the most frequently performed operation in both the Specialist (40.2%) and Resident (41.2%) groups (Table 1). Furthermore, the pathological evaluation confirmed that the groups were well-matched regarding tumor stage complexity. No significant differences were observed in the distributions of pathological T-stage (*p* = 0.331) and N-stage (*p* = 0.121) (Table 2), confirming that the residents were not exclusively assigned to early-stage or anatomically simpler cases.

### 3.3. Perioperative Outcomes and Educational Time Investment

The median operative time was significantly prolonged in the Resident group compared with the Specialist group (340 [range 210 to 725] minutes vs. 290 [range 60 to 560] minutes; median difference 50 min, 95% CI 5 to 90; *p* = 0.023) (Table 2). While statistically significant, this 50 min prolongation was considered a clinically manageable and expected consequence of intraoperative training. This extended operative duration did not translate into a delayed postoperative recovery, as the median length of hospital stay remained statistically comparable between the groups (9 [range 5 to 65] days vs. 8 [range 4 to 34] days; median difference 1 day, 95% CI 0 to 2; *p* = 0.057) (Table 2). Precise quantitative data regarding intraoperative blood loss were retrospectively unavailable in the medical records and therefore could not be analyzed.

### 3.4. Oncologic Adequacy and Surgical Safety

Crucially, the resident training process was not associated with measurable differences in the short-term outcomes reported. Regarding oncologic adequacy, no statistically significant difference in median lymph node yield was detected between the Resident and Specialist cohorts (17 vs. 16 nodes; Hodges-Lehmann median difference 2 nodes, 95% CI −1 to 5; *p* = 0.251) (Table 2). Similarly, the rates of positive surgical margins were comparable (5.9% [2/34] vs. 10.8% [11/102]; absolute risk difference −4.9%, 95% CI −14.8% to 5.0%; *p* = 0.517) (Table 2).

Regarding postoperative surgical safety, anastomotic leakage was evaluated in 107 patients at risk (excluding patients who underwent abdominoperineal resections or subtotal/total colectomies with end stomas). The overall anastomotic leakage rate was 3.7% (4/107), and there was no significant difference in the incidence of this major complication between the Resident (3.4% [1/29]) and Specialist (3.8% [3/78]) groups (absolute risk difference −0.4%, 95% CI −8.3% to 7.5%; *p* = 1.000) (Table 2). Among the four patients who developed anastomotic leakage, two required reoperation (one in the Resident group and one in the Specialist group), while the other two were successfully managed conservatively with prolonged hospital observation. There were no 30-day hospital readmissions or 30-day mortalities observed in either cohort.

## 4. Discussion

The evolution of minimally invasive techniques has fundamentally reshaped surgical training paradigms, establishing advanced laparoscopic skills as an indispensable component of residency curricula. However, the feasibility of senior residents performing technically demanding procedures such as Laparoscopic Colorectal Surgery (LCS)—remains a subject of debate [4,5]. Large-scale national databases, such as the American College of Surgeons National Surgical Quality Improvement Program (ACS-NSQIP), suggest that resident involvement is generally safe [9,10]. The primary contribution of this study to the literature is that it definitively suggests that surgical safety and short-term oncological adequacy can be maintained under a structured mentorship model. By using a defined cohort and excluding certain confounders, attempts were made to reduce selection bias.

Obesity is a well-established independent risk factor that complicates laparoscopic pelvic dissection. Although a high Body Mass Index (BMI) intensifies technical difficulty, it has been reported that patient safety can be preserved under rigorous expert supervision [11]. In the present cohort, no statistically significant variance was observed between the Specialist and Resident groups regarding BMI (*p* = 0.629)**,** distributions of advanced-stage (T3–T4) tumors (*p* = 0.331), and node-positive disease (pN1-N2) (*p* = 0.121). Consequently, the significantly prolonged operative time observed in the Resident group (340 vs. 290 min; *p* = 0.023) likely reflects the meticulous execution of core surgical steps (e.g., high vascular ligation, splenic flexure mobilization, and deep pelvic dissection) alongside the instructive dialog inherent to intraoperative mentoring, rather than anatomical disadvantages.

Consistent with the findings of this study, a large-scale analysis of the ACS-NSQIP database by de Geus et al. demonstrated that while resident involvement in laparoscopic colectomies significantly prolongs operative time, it does not negatively impact the rates of major surgical complications or reoperations [9,10,12]. Furthermore, the literature suggests that supervised resident involvement is safe not only in elective oncological resections but also in technically demanding emergent procedures such as perforated diverticulitis [13]. In this single-center series, the 50 min extension was considered an acceptable educational time investment and did not negatively impact short-term clinical recovery; indeed, no detrimental differences were observed regarding the median length of hospital stay (*p* = 0.057) or the incidence of anastomotic leakage (*p* = 1.000). Among the patients strictly at risk for leakage, leak rates were low in both groups, with no statistically significant difference detected; however, the low event rate (3.4% vs. 3.8%) limits statistical precision.

Therapeutic success in curative-intent colorectal oncology is contingent upon adherence to oncologic principles, particularly the integrity of Total Mesorectal Excision (TME), surgical margin negativity and adequate locoregional lymphadenectomy. Structured surgical training programs have previously been shown not to compromise these oncological standards [8,14]. Corroborating this paradigm, the comparative oncological outcomes demonstrated that the median number of harvested lymph nodes in the Resident group (17 nodes) was statistically equivalent to that of the Specialist cohort (16 nodes; *p* = 0.251), exceeding the clinically recommended threshold of 12 nodes. Similarly, no increased risk was observed in the Resident group regarding positive surgical margin (R1/R2) rates (5.9% vs. 10.8%; *p* = 0.517). While short-term surrogate markers such as lymph node yield and margin negativity cannot definitively prove long-term oncologic adequacy, these data show an association suggesting that the supervising surgeon effectively maintains oncological radicality by providing active guidance during critical milestones of the procedure.

High case volumes providing a uniform training model afford referral teaching hospitals the opportunity to implement structured strategies that allow residents to safely reach their learning curve plateau [15]. As recently demonstrated by Chern et al., coupling a modular, step-by-step laparoscopic training approach with the direct supervision of experienced specialists facilitates the acquisition of complex technical skills without increasing the risk of postoperative adverse outcomes [15,16].

The necessity of structured training programs in advanced minimally invasive surgery is increasingly recognized across surgical disciplines. In the present study, resident education was strictly governed by the Turkish Surgical Society guidelines and the Ministry of Health’s Board of Medical Specialties (TUKMOS) General Surgery Core Curriculum (*Genel Cerrahi Uzmanlık Eğitimi Çekirdek Müfredatı*) [17]. While some surgical specialties utilize standalone certification programs (such as the GESEA in gynecology), general surgery residency in Turkiye does not require an external certification for laparoscopy. Instead, the national TUKMOS curriculum inherently integrates basic and advanced laparoscopic competency milestones directly into the standard 5-year residency timeline. Trainees must achieve these predefined institutional competencies before they are permitted to perform complex pelvic operations under supervision. Furthermore, the feasibility of integrating advanced minimally invasive techniques into resident education is not exclusive to general surgery. Similar supervised training paradigms have been successfully implemented in other disciplines. For instance, in gynecology and urology, studies have consistently demonstrated that senior residents can safely perform complex laparoscopic and robotic procedures—such as hysterectomies and prostatectomies—without compromising patient safety or operative outcomes [18,19]. These cross-disciplinary findings corroborate the premise that structured, step-by-step mentorship within a standardized core curriculum mitigates the risks associated with the learning curve of advanced minimally invasive surgery.

This study possesses several limitations. First, its retrospective, single-center design and the relatively small size of the resident cohort limit its external generalizability. The small sample size also introduces a potential Type II error for detecting differences in rare complications such as anastomotic leakage. Furthermore, although attempts were made to reduce selection bias by matching the groups according to BMI and tumor stage, the decision to allow a resident to operate as the primary surgeon was ultimately at the attending surgeon’s discretion. The exact intraoperative blood loss volumes were retrospectively unavailable, and the deliberate exclusion of converted cases to maintain initial cohort homogeneity inherently introduces a favorable selection bias. Because converted cases were excluded, these findings apply solely to laparoscopic procedures completed without conversion and may not reflect outcomes across all attempted laparoscopic colorectal resections. Furthermore, residual confounding by unmeasured clinical, anatomical, treatment-related, and operator-level factors cannot be excluded. Finally, validated educational assessment tools or learning curve evaluations were not utilized, limiting the ability to objectively quantify resident competency acquisition.

## 5. Conclusions

By using a defined cohort and excluding certain confounders, attempts were made to reduce selection bias and demonstrate comparable short-term outcomes between training and specialist cases. The findings of this study suggest that Laparoscopic Colorectal Surgery performed by senior general surgery residents under the direct, intraoperative supervision of experienced specialists is associated with no material difference in the measured short-term outcomes, within this single-center supervised training model and the eligibility criteria used. The prolongation of operative time associated with resident participation appears to be an acceptable aspect of surgical education. Future multicenter prospective studies with larger sample sizes, formal resident competency assessments, and long-term oncologic follow-up are recommended to confirm the safety and educational value of resident-performed Laparoscopic Colorectal Surgery.

## Figures and Tables

**Table 1:** Patient demographics and distribution of surgical procedures.

Variables	Specialist (*n* = 102)	Resident (*n* = 34)	*p*-Value
Age (Years, Mean ± SD)	61.8 ± 10.7	62.2 ± 11.8	0.868
BMI (kg/m^2^, Median [Min–Max])	26.33 [18.3–39.3]	26.37 [16.2–36.7]	0.629
Gender (*n*, %)			0.115
- Male	72 (70.6%)	19 (55.9%)	
- Female	30 (29.4%)	15 (44.1%)	
Procedure Type (*n*, %)			0.582
- Right Hemicolectomy	24 (23.5%)	9 (26.5%)	
- Left Hemicolectomy	5 (4.9%)	1 (2.9%)	
- Anterior Resection (AR)	7 (6.9%)	3 (8.8%)	
- Low Anterior Resection (LAR)	41 (40.2%)	14 (41.2%)	
- Abdominoperineal Resection (Miles)	24 (23.5%)	5 (14.7%)	
- Subtotal/Total Colectomy	1 (1.0%)	2 (5.9%)	

SD: Standard Deviation, BMI: Body Mass Index.

**Table 2:** Comparison of perioperative, postoperative, and oncologic outcomes.

Variables	Specialist (*n* = 102)	Resident (*n* = 34)	*p*-Value
Operative Time (min, Median [Min–Max])	290 [60–560]	340 [210–725]	**0.023** *
Length of Stay (days, Median [Min–Max])	8 [4–34]	9 [5–65]	0.057
Lymph Node Count (Median [Min–Max])	16 [1–48]	17 [4–109]	0.251
Surgical Margin Status (*n*, %)			0.517
- Negative	91 (89.2%)	32 (94.1%)	
- Positive	11 (10.8%)	2 (5.9%)	
Anastomotic Leakage (*n*, %) ^†^			1.000
- Absent	75 (96.2%)	28 (96.6%)	
- Present	3 (3.8%)	1 (3.4%)	
Pathologic T Stage (pT) (*n*, %)			0.331
- T0	8 (7.8%)	1 (2.9%)	
- T1/T2	39 (38.3%)	8 (23.5%)	
- T3/T4	55 (53.9%)	25 (73.5%)	
Pathologic N Stage (pN) (*n*, %)			0.121
- N0	70 (68.6%)	17 (50.0%)	
- N1/N2	32 (31.4%)	17 (50.0%)	

* *p* < 0.05 was considered statistically significant; ^†^ Calculated for 107 patients at risk (Specialist *n* = 78; Resident *n* = 29), excluding abdominoperineal resections and subtotal/total colectomies with end stomas. Bold values indicate statistical significance (*p* < 0.05).

## Data Availability

The raw data supporting the conclusions of this article will be made available by the authors on request.

## Abbreviations

The following abbreviations are used in this manuscript:

ACS-NSQIP	American College of Surgeons National Surgical Quality Improvement Program
AR	Anterior Resection
BMI	Body Mass Index
EPV	Events-Per-Variable
IRB	Institutional Review Board
LAR	Low Anterior Resection
LCS	Laparoscopic Colorectal Surgery
SD	Standard Deviation
TME	Total Mesorectal Excision
